# Genomic index selection provides a pragmatic framework for setting and refining multi-objective breeding targets in *Miscanthus*

**DOI:** 10.1093/aob/mcy187

**Published:** 2018-10-23

**Authors:** Gancho T Slavov, Christopher L Davey, Maurice Bosch, Paul R H Robson, Iain S Donnison, Ian J Mackay

**Affiliations:** 1 Computational & Analytical Sciences Department, Rothamsted Research, Harpenden, Hertfordshire, UK; 2 Institute of Biological, Environmental and Rural Sciences, Aberystwyth University, Aberystwyth, UK; 3 IMPlant Consultancy Limited, Chelmsford, UK

**Keywords:** Selection indices, genomic selection, breeding objectives, economic values, correlated responses, *Miscanthus sinensis*

## Abstract

**Background:**

*Miscanthus* has potential as a biomass crop but the development of varieties that are consistently superior to the natural hybrid *M. × giganteus* has been challenging, presumably because of strong G × E interactions and poor knowledge of the complex genetic architectures of traits underlying biomass productivity and climatic adaptation. While linkage and association mapping studies are starting to generate long lists of candidate regions and even individual genes, it seems unlikely that this information can be translated into effective marker-assisted selection for the needs of breeding programmes. Genomic selection has emerged as a viable alternative, and prediction accuracies are moderate across a range of phenological and morphometric traits in *Miscanthus*, though relatively low for biomass yield *per se*.

**Methods:**

We have previously proposed a combination of index selection and genomic prediction as a way of overcoming the limitations imposed by the inherent complexity of biomass yield. Here we extend this approach and illustrate its potential to achieve multiple breeding targets simultaneously, in the absence of *a priori* knowledge about their relative economic importance, while also monitoring correlated selection responses for non-target traits. We evaluate two hypothetical scenarios of increasing biomass yield by 20 % within a single round of selection. In the first scenario, this is achieved in combination with delaying flowering by 44 d (roughly 20 %), whereas, in the second, increased yield is targeted jointly with reduced lignin (–5 %) and increased cellulose (+5 %) content, relative to current average levels in the breeding population.

**Key Results:**

In both scenarios, the objectives were achieved efficiently (selection intensities corresponding to keeping the best 20 and 4 % of genotypes, respectively). However, the outcomes were strikingly different in terms of correlated responses, and the relative economic values (i.e. value per unit of change in each trait compared with that for biomass yield) of secondary traits included in selection indices varied considerably.

**Conclusions:**

Although these calculations rely on multiple assumptions, they highlight the need to evaluate breeding objectives and explicitly consider correlated responses *in silico*, prior to committing extensive resources. The proposed approach is broadly applicable for this purpose and can readily incorporate high-throughput phenotyping data as part of integrated breeding platforms.

## SIMULTANEOUS IMPROVEMENTS IN MULTIPLE TRAITS ARE NEEDED FOR *MISCANTHUS* TO BECOME A WIDELY GROWN BIOMASS CROP

Despite relatively poor uptake so far, the potential of *Miscanthus* as a biomass crop is high, and many technical barriers to its broader adoption have largely been resolved (Clifton‐Brown *et al.*, 2017). However, several major challenges remain, including the need to reduce the cost of establishment further and to develop varieties with consistently higher yields than the sterile triploid *M.* × *giganteus* clones, which are currently the only commercially available option. The potential for *Miscanthus* as a biomass crop in Europe, the USA and in Asia has been discussed over the last two decades, and several studies highlight the need for cold and drought tolerance, in particular, to be improved (Lewandowski *et al.*, 2000; Heaton *et al.*, 2004; Richter *et al.*, 2008; Chung and Kim, 2012; Liu *et al.*, 2012). Improvements in abiotic stress tolerance are also needed to mitigate the potential impact of climate change and to allow biomass crops to generate high yields on marginal lands. For example, Hastings *et al.* (2009) predicted an 80 % decline in energy production from *M.* × *giganteus* due to a reduction in seasonal water availability due to climate change in Europe up to 2080. Improved tolerance to single or combinations of abiotic stresses will allow *Miscanthus* to be grown increasingly on areas of marginal land (Stavridou *et al*., 2017, 2019), thus preventing potential competition with food crops (Valentine *et al.*, 2012).

Although significant progress has been made already, much remains to be learned about the abiotic stress resilience of *Miscanthus*, which will be a crucial factor for its successful deployment in a broad range of environments. Unusually for a C_4_ crop, *Miscanthus* exhibits cold-tolerant photosynthesis (Naidu *et al.*, 2003; Long and Spence, 2013), although there remains significant potential to increase yield by up to 25 % by extending the season through improved cold tolerance (Farrell *et al.*, 2006). When a small number of chilling-tolerant and chilling-sensitive genotypes were compared under field conditions, there appeared to be a trade-off between high photosynthesis and chilling tolerance. The commercial clone *M.* × *giganteus* displayed both early season chilling tolerance and, later in the season, high photosynthesis more typical of the chilling-sensitive genotypes (Fonteyne *et al.*, 2018). Drought tolerance is another major opportunity for improvement as *M.* × *giganteus* is reported to have poor water-use efficiency, and yield is strongly impacted by water stress (Clifton-Brown and Lewandowski, 2000; Ings *et al.*, 2013). However, the genus comprises highly diverse species that have developed across different habitats in Asia (Clifton-Brown *et al.*, 2008; Sacks *et al.*, 2013), and such diversity may hold the key to overcoming yield limitations due to abiotic stress. Performance under water stress treatment has been related to meteorological data from the site of origin (Weng, 1993; Malinowska *et al.*, 2017) and illustrates the potential to combine the useful variation within diverse *Miscanthus* ecotypes to produce superior germplasm.

Finally, there is also tremendous potential for manipulating biomass quality, particularly cell wall structure and composition (Slavov *et al.*, 2013*a*; Bhatia *et al.*, 2017). Given the complex nature of biomass recalcitrance to deconstruction, dictated by the relative abundances and interactions of the cell wall components within the cell wall matrix, it is difficult to predict recalcitrance based on single compositional traits. Nevertheless, lignin is commonly regarded as one of the main factors impeding saccharification by enzymatic hydrolysis as it prevents enzymes accessing the hemicellulose and cellulose in plant cell walls (Zeng *et al.*, 2014). Indeed, reduction in lignin content by various methods resulted in improved biomass digestibility and reduced recalcitrance (Welker *et al.*, 2015). Increasing cellulose content represents another potential breeding target for improving biomass quality as this would increase the proportion of easily fermentable glucose monosaccharides (Furtado *et al.*, 2014; Bhatia *et al.*, 2017). It is important to note that biomass quality parameters depend on the intended technology for lignocellulose conversion (van der Weijde *et al.*, 2017*b*; da Costa *et al.*, 2019). For instance, while a low lignin content may improve enzymatic conversion, its high energy density means that a high lignin content is favourable for thermochemical conversion (Welker *et al.*, 2015). Other biomass quality parameters that influence thermochemical conversion (such as combustion, gasification and pyrolysis) include ash, mineral and moisture content (Tanger *et al.*, 2013). Interestingly, previous studies have shown organ-specific abundance, distribution, composition and ornamentation of cell wall polymers (e.g. acetylation, arabinosylation and feruloylation of arabinoxylans), affecting sugar release differently (da Costa *et al*., 2014, 2017). Hence, besides targeting cell wall features directly, biomass quality can possibly also be manipulated by allometry (i.e. the relative proportions of leaf vs. stem biomass) in order to reduce recalcitrance to bioconversion or increase the biomass properties for thermochemical conversion. Furthermore, drought treatment also impacts cell wall composition and, consequently, how the biomass is processed. For example, in a study of 50 diverse *Miscanthus* genotypes, drought tolerance and cell wall composition were only weakly correlated, suggesting that the potential exists for both traits to be improved independently (van der Weijde *et al.*, 2017*a*). More generally, future domestication and breeding efforts are likely to target the joint improvement of biomass yield, cell wall composition and abiotic stress resilience.

## LINKAGE AND ASSOCIATION MAPPING STUDIES CAN PROVIDE INSTRUCTIVE CASE STUDIES, BUT ARE UNLIKELY TO TRANSFORM BREEDING APPROACHES

Recent advances in sequencing and genotyping technology (Davey *et al.*, 2011) have made high-density linkage and association mapping studies affordable across a range of food (Buckler *et al.*, 2009; Bentley *et al.*, 2014) and energy crops (Evans *et al.*, 2014; Hallingback *et al.*, 2016), including *Miscanthus* (Slavov *et al.*, 2014; Gifford *et al.*, 2015; Clark *et al.*, 2016; Dong *et al.*, 2018). While these studies have identified numerous candidate regions and individual genes, it seems unlikely that this information can be translated into effective marker-assisted selection (MAS) for the needs of breeding programmes. One reason for this is that mapping populations developed previously for *Miscanthus* (typically *n* = 100–200) were too small (Cockram and Mackay, 2018) relative to the presumed high complexity of trait genetic architectures. Consequently, many of the associations and quantitative trait loci (QTLs) detected so far are likely to be false positives [i.e. because low statistical power tends also to result in higher rates of false positives (Button *et al.*, 2013)], while the naïve effect size estimates of all detected QTLs/associations are inflated by as much as an order of magnitude (Beavis, 1998; Allison *et al.*, 2002). Although several larger mapping populations (*n* = 500–1000) have recently become available for *Miscanthus* (G. Slavov and C. Davey, unpubl. data; P. Robson, unpubl. data; L. Clark and E. Sacks, pers. comm.), statistical power will remain extremely low for the foreseeable future (Visscher *et al.*, 2017). Another, less tractable problem is that combining favourable alleles even for as few as 30 unlinked QTLs is a major logistical challenge (Bernardo, 2016), while some quantitative traits may be controlled by thousands, or even tens of thousands, of loci (Boyle *et al.*, 2017; Visscher *et al.*, 2017). Thus, MAS may be a viable breeding approach for traits that are controlled by major loci (e.g. disease resistance), but probably not for most quantitative traits (Bernardo, 2016).

Genomic prediction [also referred to as genomic selection (GS): trait prediction from a genome-wide set of markers, without necessarily testing for significant marker–trait associations (Meuwissen *et al.*, 2001)] currently appears to be an effective alternative to MAS, although relatively few examples of the successful implementation of GS in actual plant breeding programmes have been published (Bernardo, 2016). Based on a pilot-scale study in a population of 138 *M. sinensis* genotypes, the accuracy of genomic prediction was moderate across a range of phenological and morphometric traits, though very low for biomass yield *per se* (Slavov *et al.*, 2014). In a recent follow-up study (Davey *et al.*, 2017), we demonstrated that this limitation can easily be overcome by capturing the genetic correlations between biomass yield and individual phenological and morphometric traits. Here we extend this approach and illustrate its potential simultaneously to achieve multiple breeding targets in the absence of *a priori* knowledge about their relative economic importance, while also monitoring correlated selection responses for non-target traits.

## GENOMIC INDEX SELECTION CAN BE USED TO EVALUATE BREEDING TARGETS *IN SILICO*

### Methodology

Dating back nearly a century (Smith, 1936), the idea of a selection index is optimally to combine all available information about the genetic worth (i.e. breeding value) of an individual, including information about its relatives and/or multiple traits with different heritabilities and genetic/environmental correlations (Falconer, 1989). Briefly, to construct a selection index, one needs to have estimates of the phenotypic and genetic variance–covariance matrices, as well as a quantitative measure of the relative importance of different traits (i.e. economic values). The index (*I*) is a linear combination of phenotypic trait values and is calculated for each individual (line or genotype) as:

I=b′X (1)

where *b*′ is a transposed *n* × 1 vector of weighting factors and *X* an *n* × 1 vector of phenotypic measurements for *n* traits.

The weighting factors for the index are obtained by solving:

$$b=P^{–\text{1}}Ga$$ (2)

where *P*^–1^ is the inverse of the *n* × *n* phenotypic variance–covariance matrix, *G* is the *n* × *n* additive genetic variance–covariance matrix and *a* is an *n* × 1 vector of economic values per unit of change for each trait. Selection for the index is then expected to be more efficient than selecting for individual traits in successive generations (tandem selection) or independently culling individuals based on standards for each trait.

In a recent study (Davey *et al.*, 2017), we demonstrated that selection indices targeting biomass yield can effectively exploit the genetic correlations of this highly composite trait with individual phenological and morphometric traits to increase the accuracy of genomic prediction by an order of magnitude, while also increasing the expected response relative to selection for biomass yield alone, as expected from quantitative genetics theory (Falconer, 1989; Mackay *et al.*, 2015). To achieve this, we assigned an economic value of 1 to biomass yield and 0 to all other traits included in any given selection index (Mackay *et al.*, 2015). This approach was effective for improving the accuracy of genomic prediction, but does not fully exploit the conceptual strength of index selection (i.e. simultaneous improvement of multiple valuable traits). Because the objective assignment of economic values is challenging for many traits, a potentially more useful alternative approach is to specify desired selection gains for all *n* or a sub-set of *m* traits (Yamada *et al.*, 1975). In the simple case of *n* = *m*, and when the traits of breeding interest are the same as the traits that are measured, the weighting factors for the index can be calculated as:

$$b=G^{–\text{1}}Q$$ (3)

where *Q* is an *n* × 1 vector of desired genetic gains and *G*^–1^ is the inverse of the *n* × *n* additive genetic variance–covariance matrix, which is typically estimated based on quantitative genetic analyses of phenotypic data from relatives (Falconer, 1989). More importantly, this approach can also be applied when the breeder is only able to set desired genetic gains for a sub-set of the traits of interest (e.g. because of insufficient information to inform decisions). In that case (*n* > *m*), after simplifying equation 13 of Yamada *et al.* (1975) by setting the relationship matrix to unity (i.e. assuming that selection candidates are phenotyped directly), the *n* weighting factors for the selection index are calculated as:

$$b=P^{-1}G^{*}[{G^{*}}^{\prime }P^{-1}G^{*}{]}^{-1}Q$$ (4)

where *Q* is an *m* × 1 vector of desired genetic gains, G* is a non-invertible *n* × *m* matrix, which is derived from *G* by only keeping the *m* columns for the traits represented in *Q*, and *P*^–1^ is the inverse of the *n* × *n* phenotypic variance–covariance matrix. Rearranging eqn (2), economic values corresponding to the desired genetic gains in *Q* can then be calculated as:

$$a=G^{–\text{1}}Pb$$ (5)

Thus, breeders are given the option of assessing the changes in relative economic values resulting from the choice of desired genetic gains, as well as to evaluate the *n* × 1 vector of correlated responses (Q*) for traits for which desired genetic gains had not been specified by calculating:

Q* =Gb (6)

Finally, assuming that the intensity of selection (*i*) required to achieve the desired genetic gains is equal to the standard deviation of the selection index *σ*_1_ [i.e. the breeding objective is to be achieved in a single generation, Yamada *et al.* (1975)], *i* can be calculated as:

$$i=\sigma_{\text{I}}=\sqrt{\left(b^{\prime }Pb\right)}$$ (7)

### Illustration through two genomic index selection scenarios in *Miscanthus*

The framework described above (see ‘Methodology’) allows breeders to assess different multi-objective selection scenarios *in silico*, without complete knowledge about the economic values of target traits. For example, eqns (4)–(6) make it possible to set breeding targets for only a small sub-set of traits (i.e. those for which desired genetic changes can be justified), evaluate the economic values associated with setting these targets and assess correlated responses for other traits of interest. Furthermore, if breeders are not satisfied with some of the economic values and/or expected correlated responses, selection targets can be modified iteratively until an acceptable solution is found.

We illustrate the flexibility of this approach through two hypothetical scenarios based on real data from *Miscanthus sinensis* (Slavov *et al*., 2013*b*, 2014; Davey *et al.*, 2017) and variance–covariance matrices estimated using molecular markers. In addition to dry biomass yield (DryMatter) and moisture content, the traits of interest include two phenological, nine morphometric (i.e. excluding the less easily interpretable combined StatureCategory trait used in earlier studies) and three cell wall composition measurements (Table 1). The primary breeding objective in both scenarios was to increase dry biomass yield by 20 % relative to the mean for the current population. The first scenario (S1) also aimed to delay flowering by 44 d (approx. 20 %) to better take advantage of the growing season (Jensen *et al.*, 2011), while also exploiting the genetic correlation (*r* = 0.19) between flowering time and yield (Slavov *et al.*, 2014). In contrast, the second scenario (S2) targeted changes in cell wall composition (i.e. a 5 % reduction in lignin and a 5 % increase in cellulose), without aiming to alter phenology. These cell wall composition changes may be expected to increase the yields of biomass conversion to ethanol, with the reduction in lignin alone expected to result in roughly 5 % higher ethanol yields per unit of dry biomass (da Costa *et al.*, 2014).

**Table 1.** Definitions, broad-sense heritabilities and genomic predictive abilities for 16 traits measured in 138 *M. sinensis* genotypes

Trait*	Definition	*H* ^2†^	*r*_*Sorg*_ (s.d.)^‡^
**Phenology**			
DOYFS1.9	Date of flowering stage 1: day of year when the first flag leaf emerged	0.89	0.76 (0.02)
AvgeSen.9	Average senescence score (0–10) throughout the growing season	0.83	0.64 (0.01)
**Morphology/biomass**			
BaseDiameter.9	Largest plant diameter measured at ground level (mm)	0.52	0.27 (0.05)
DryMatter.9	Estimated total dry weight per plant (g)	0.54	0.06 (0.05)
LeafLength.7	Ligule to tip length along the central vein of the youngest leaf with a ligule (cm)	0.65	0.67 (0.01)
LeafWidth.7	Blade width at half-leaf length for the leaf used to measure LeafLength (cm)	0.64	0.52 (0.02)
MaxCanopyHeight.9	Height from the ground to the point of ‘inflection’ of the majority of leaves (cm)	0.77	0.35 (0.03)
Moisture.9	Estimated moisture content based on a sub-sample (%)	0.59	0.70 (0.01)
StatureLeafAngle.7	Three-category score reflecting leaf angle relative to the vertical (0 = vertical, 0.5 = intermediate, 1 = horizontal)	0.50	0.46 (0.03)
StatureStemAngle.7	Four-category score reflecting stem angle relative to the vertical (1 = upright stems, 2 = stems inclined up to 30° from the vertical, 3 = stems inclined up to 60° from the vertical, 4 = stems inclined up to 90° from the vertical)	0.48	0.37 (0.02)
StemDiameter.9	Diameter 10–15 cm from the ground of a randomly chosen stem (mm)	0.60	0.51 (0.03)
TallestStem.9	Length of the tallest stem (cm)	0.88	0.65 (0.01)
TransectCount.9	Number of stems with ≥50 % canopy height across the middle of the plant	0.51	0.17 (0.04)
**Cell wall composition**			
Cellulose.8	Gravimetrically measured cellulose content (% d. wt)	0.79	0.62 (0.02)
Hemicellulose.8	Gravimetrically measured hemicellulose content (% d. wt)	0.60	0.25 (0.03)
Lignin.8	Gravimetrically measured lignin content (% d. wt)	0.66	0.43 (0.02)

*Trait: phenotypic traits measured in 2007 (.7), 2008 (.8) or 2009 (.9) (i.e. after two, three or four growing seasons, respectively). Detailed phenotyping protocols were described by Slavov *et al.* (2013*b*).

^†^ *H* ^2^: broad-sense heritability (Slavov *et al.*, 2014).

^‡^ *r*_*Sorg*_ (s.d.): average predictive ability and standard deviation across 100 random 10-fold cross-validations based on 53 174 single-nucleotide variants obtained from alignments to the *Sorghum bicolor* genome (Slavov *et al.*, 2014).

We made slight modifications to the approach used to estimate genetic and phenotypic variance–covariance matrices in our previous study (Davey *et al.*, 2017), in which selection indices were used primarily as a way to increase the genomic predictive ability of dry biomass yield. The additive genetic variance–covariance matrix (*G*) for the 16 traits was estimated from approx. 53 000 single-nucleotide variants generated using a RAD-Seq genotyping approach and alignments to the *Sorghum bicolor* genome (Slavov *et al.*, 2014). This was done by fitting a multi-trait GBLUP model (Jia and Jannink, 2012) using the MTM R package (Cuevas *et al.*, 2017) with unstructured covariance matrices to the best linear unbiased estimator (BLUE) values for the 16 traits. Because the emphasis of this analysis was the estimation of *G* from a genome-wide set of markers, we used BLUEs instead of best linear unbiased predictors (BLUPs), which were preferred in our previous studies (Slavov *et al.*, 2014; Davey *et al.*, 2017), to avoid the undesirable shrinkage and consequent underestimation of *G* associated with the BLUP approach (Garrick *et al.*, 2009). Following a burn-in of 1000 iterations, we ran 10 000 iterations of the Gibbs sampler in MTM, keeping every fifth iteration (thin = 5) to reduce auto-correlation. The phenotypic variance–covariance matrix (*P*) was calculated as the sum of the genetic and residual variance–covariance matrices estimated using MTM (Supplementary Data File S1). Weighting factors for the selection indices corresponding to each scenario were then calculated using eqn 4, while economic values, expected correlated responses and selection intensities were estimated using eqns (5), (6) and (7) respectively. All input data sets and scripts for these analyses are available at github.com/ChrisDaveyCymru/genomic_index_selection.

**Fig. 1.**
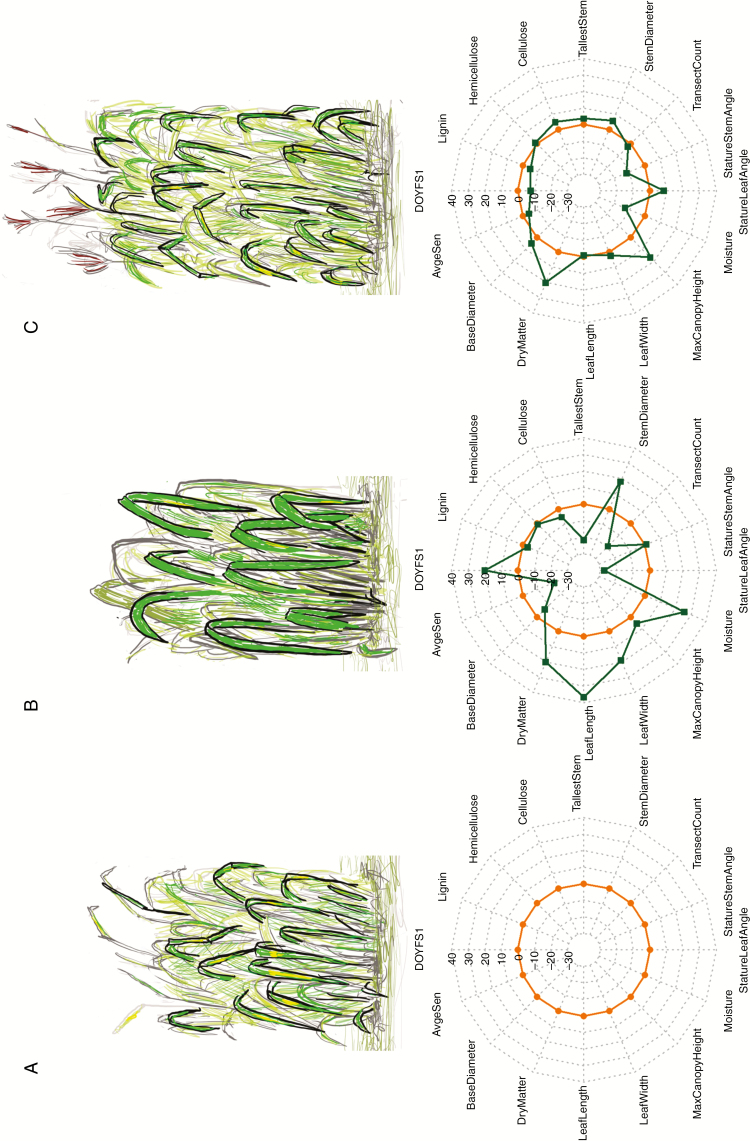
Quantitative (bottom) and schematic (top) representation of current population mean values (A) for 16 phenotypic traits (Table 1) and outcomes from genomic index selection scenarios S1 (increase biomass yield by 20 % and delay flowering by 44 d; B) and S2 (increase biomass yield by 20 %, while also increasing cellulose content by 5 % and reducing lignin content by 5 %; C). The orange line in all radar charts is set to 0 and corresponds to the baseline (i.e. current population mean values; Table 2). The green lines in (B) and (C) correspond to the relative changes (%) for the 16 traits (Table 2).

The desired genetic gains in both scenarios were achievable within a single round of selection with relatively low intensity [*i* =1.41 and 2.15, corresponding to keeping the best 20 and 4 % of genotypes in S1 and S2, respectively (Falconer, 1989)]. However, the outcomes were strikingly different in terms of correlated responses (Table 2; Fig. 1; Supplementary Data File S1). As expected from the pre-specified genetic gains, biomass yield in S1 was increased by 20 %, while flowering was delayed by 44 d (20 %). Unfavourably, this also resulted in 21 % lower average senescence and 26 % higher biomass moisture content, as expected from the strong genetic correlations of these traits with flowering time (*r*_g_ > 0.8, Supplementary Data File S1). However, somewhat surprisingly, given the moderate genetic correlation between stem/canopy height and biomass yield (Slavov *et al.*, 2014; Supplementary Data File S1), the canopy height increased modestly (5 %), whereas the length of the tallest stem was reduced by 22 %. Furthermore, both BaseDiameter (i.e. a measure of the overall area occupied by each plant) and TransectCount (i.e. a correlate of the total number of stems) were reduced by 7 and 19 %, respectively. This was counterbalanced by substantial increases in stem diameter (18 %), leaf width (19 %) and particularly leaf length (37 %) (i.e. leaves can form a substantial proportion of final biomass yields; da Costa *et al.*, 2014). Leaf orientation also changed considerably, with leaf angle shifting by 28 % towards the vertical. Finally, cellulose, hemicellulose and lignin contents were all reduced (by approx. 5, 1 and 3 %, respectively).

**Table 2.** Absolute (Δ) and relative (%) changes in trait values resulting from two contrasting genomic index selection scenarios (see text and Supplementary Data File S1 for additional parameter estimates)

Trait (unit)*	Current mean	ΔS1 (%)^†^	ΔS2 (%)^‡^
**Phenology**			
DOYFS1.9 (d)	221.21	44.00 (19.9)	–17.29 (–7.8)
AvgeSen.9 (0–10)	7.34	–1.51 (–20.6)	–0.30 (–4.1)
**Morphology/biomass**			
BaseDiameter.9 (mm)	398.72	–26.70 (–6.7)	18.94 (4.8)
DryMatter.9 (g)	1065.47	213.00 (20.0)	213.00 (20.0)
LeafLength.7 (cm)	52.43	19.19 (36.6)	–0.55 (–1.0)
LeafWidth.7 (cm)	1.42	0.27 (18.9)	0.03 (2.4)
MaxCanopyHeight.9 (cm)	144.67	7.54 (5.2)	24.44 (16.9)
Moisture.9 (%)	30.65	7.88 (25.7)	–3.98 (–13.0)
StatureLeafAngle.7 (0–1)	0.66	–0.18 (–27.6)	0.06 (8.4)
StatureStemAngle.7 (1–4)	1.97	0.02 (1.0)	–0.24 (–12.0)
StemDiameter.9 (mm)	5.37	0.97 (18.1)	0.31 (5.8)
TallestStem.9 (cm)	176.82	–38.21 (–21.6)	6.53 (3.7)
TransectCount.9 (count)	27.63	–5.34 (–19.3)	–0.61 (–2.2)
**Cell wall composition**			
Cellulose.8 (%)	42.14	–2.17 (–5.2)	2.10 (5.0)
Hemicellulose.8 (%)	32.81	–0.22 (–0.7)	0.39 (1.2)
Lignin.8 (%)	8.95	–0.29 (–3.3)	–0.45 (–5.0)

Weighting factors for the selection indices corresponding to each scenario were calculated using eqn (4), while economic values, expected correlated responses and selection intensities were estimated using eqns (5), (6) and (7), respectively.

*Trait (unit): phenotypic traits as defined in Table 1. Detailed phenotyping protocols were described by Slavov *et al.* (2013*b*).

^†^Breeding scenario with objectives: (1) increase yield (DryMatter) by 20 % and (2) delay flowering (DOYFS1) by 44 d (approx. 20 %). The genetic gains listed in the table can be achieved in a single round of selection with intensity = 1.41 (i.e. selecting the top 20 % of lines based on genomic estimated aggregate breeding values). The relative economic values per unit of change for the traits in the selection index are DOYFS1:DryMatter = 2.2:1.

^‡^Breeding scenario with objectives: (1) increase yield by 20 %, (2) increase cellulose content by 5 % and (3) reduce lignin by 5 %. The genetic gains listed in the table can be achieved in a single round of selection with intensity = 2.15 (i.e. selecting the top 4 % of lines based on genomic estimated aggregate breeding values). The relative economic values per unit of change for the traits in the selection index are Lignin:Cellulose:DryMatter = –180:72:1.

In contrast, the mean flowering time under S2 was shifted 17 d earlier (8 %) compared with the current population mean, with a surprising (i.e. inconsistent with the sign of the genetic correlation) slight decrease in senescence (4 %), but also with a substantial reduction in biomass moisture content (13 %). Furthermore, canopy height was increased considerably (17 %), while there were also modest increases in the length of the tallest stem, and base and stem diameters (4, 5 and 6 %, respectively). Leaf and stem orientation both changed, with leaf angle shifting by 8 % towards the horizontal, but stems becoming 12 % more upright. Finally, changes in cellulose and lignin contents were as specified by desired genetic changes (an increase and a reduction of 5 %, respectively), while there was also a small increase (1 %) in hemicellulose content.

The relative economic values of secondary traits included in selection indices varied considerably (Table 2; Supplementary Data File S1). In S1, for example, the economic value of delaying flowering by 1 d was only 2.2 times higher than that of increasing biomass yield by 1 g per plant (Table 2). Given that plants were grown on a 1.5 × 1.5 m grid (4444 plants ha^–1^), the delay of flowering by 44 d in S1 translates to the equivalent of an increase in dry biomass by 0.43 t ha^–1^ (i.e. 4444 × 44 × 2.2/1000/1000), or roughly €30 ha^–1^, if a price of €70 t^–1^ is assumed (Witzel and Finger, 2016). With similar assumptions, the economic values of increasing cellulose by 5 % and decreasing lignin by 5 % in S2 would be equivalent to yield increases worth approx. €112 and €280 ha^–1^, respectively (e.g. the breeding effort required to reduce lignin by 5 % would be equivalent to increasing biomass yield by 4.0 t ha^–1^, translating to €280 ha^–1^). However, the increased efficiency of bioconversion expected to result from these modifications (which is not reflected in these calculations) may substantially influence these comparisons. Ultimately, whether these economic values are acceptable will probably depend on many factors, including the expected scale of deployment of the crop, the risk of maladaptation associated with changes in phenological traits and the actual effects of changes in cell wall composition on bioconversion. However, having economic value estimates before breeding has been undertaken may be preferable to setting completely arbitrary selection targets.

Assuming that the outcomes of both scenarios are not completely satisfactory to breeders (e.g. because of the increase of moisture content in S1 and shift towards earlier flowering in S2), we modified the vector of desired genetic gains (*Q*), by setting target changes of 0 for moisture content in S1 and flowering time in S2 (Table 3; Supplementary Data File S1). As expected from the increased number of breeding targets, selection intensities for these refined scenarios were higher, but the increase was relatively small (*i* = 2.18 and 2.58, corresponding to keeping the best 3.8 and 1.3 % of genotypes in S1* and S2*, respectively). Correlated responses in the modified scenarios were generally similar to those in S1 and S2, with an even greater increase in leaf length and canopy height in S1* and an even larger reduction in moisture content in S2*. There were, however, also a few sign differences. For example, stem orientation in S1* became 26 % more vertical [i.e. compared with a slight (1 %) shift towards horizontal orientation in S1]. Similarly, changes in base diameter, number of stems (TransectCount) and leaf size (both length and width) switched signs between S2 and S2* (Table 2 vs. Table 3). Finally, the relative economic values of all secondary traits included in the original scenarios increased substantially in the modified scenarios, while the economic value of keeping moisture content unchanged in S1* translated to roughly €14 ha^–1^.

**Table 3.** Absolute (Δ) and relative (%) changes in trait values resulting from two refined genomic index selection scenarios (i.e. S1* and S2* are modified versions of S1 and S2 from Table 2; see text and Supplementary Data File S1 for additional parameter estimates)

Trait (unit)*	Current mean	ΔS1* (%)^†^	ΔS2* (%)^‡^
**Phenology**			
DOYFS1.9 (d)	221.21	44.00 (19.9)	0.00 (0.0)
AvgeSen.9 (0–10)	7.34	–1.09 (–14.9)	–0.37 (–5.1)
**Morphology/biomass**			
BaseDiameter.9 (mm)	398.72	–76.12 (–19.1)	–51.84 (–13.0)
DryMatter.9 (g)	1065.47	213.00 (20.0)	213.00 (20.0)
LeafLength.7 (cm)	52.43	27.14 (51.8)	11.50 (21.9)
LeafWidth.7 (cm)	1.42	0.11 (8.0)	–0.01 (–0.8)
MaxCanopyHeight.9 (cm)	144.67	13.85 (9.6)	25.50 (17.6)
Moisture.9 (%)	30.65	0.00 (0.0)	–6.61 (–21.6)
StatureLeafAngle.7 (0–1)	0.66	–0.17 (–25.8)	0.02 (2.5)
StatureStemAngle.7 (1–4)	1.97	–0.50 (–25.6)	–0.49 (–25.1)
StemDiameter.9 (mm)	5.37	0.28 (5.2)	0.10 (1.9)
TallestStem.9 (cm)	176.82	–37.66 (–21.3)	0.05 (0.0)
TransectCount.9 (count)	27.63	–0.75 (–2.7)	0.14 (0.5)
**Cell wall composition**			
Cellulose.8 (%)	42.14	–1.02 (–2.4)	2.1 (5.0)
Hemicellulose.8 (%)	32.81	0.05 (0.2)	0.59 (1.8)
Lignin.8 (%)	8.95	–0.22 (–2.4)	–0.45 (–5.0)

Weighting factors for the selection indices corresponding to each scenario were calculated using eqn (4), while economic values, expected correlated responses and selection intensities were estimated using eqns (5), (6) and (7), respectively.

*Trait (unit): phenotypic traits as defined in Table 1. Detailed phenotyping protocols were described by Slavov *et al.* (2013*b*).

^†^Breeding scenario with objectives: (1) increase yield (DryMatter) by 20 % and (2) delay flowering (DOYFS1) by 44 d (approx. 20 %), while keeping moisture content unchanged. The genetic gains listed in the table can be achieved in a single round of selection with intensity = 2.18 (i.e. selecting the top 3.8 % of lines based on genomic estimated aggregate breeding values). The relative economic values per unit of change for the traits in the selection index are Moisture:DOYFS1:DryMatter = –45:9:1;

^‡^Breeding scenario with objectives: (1) increase yield by 20 %, (2) increase cellulose content by 5 % and (3) reduce lignin by 5 %, while keeping flowering time unchanged. The genetic gains listed in the table can be achieved in a single round of selection with intensity = 2.58 (i.e. selecting the top 1.3 % of lines based on genomic estimated aggregate breeding values). The relative economic values per unit of change for the traits in the selection index are *C*ellulose:Lignin:DOYFS1:DryMatter = 518:–318:25:1.

### Implications

The analytical framework discussed above can be used to evaluate rapidly numerous scenarios with different breeding objectives, explicitly considering correlated responses and relative economic values *in silico*, prior to committing extensive resources. The proposed approach is broadly applicable for this purpose and can readily incorporate high-throughput phenotyping data as part of integrated breeding platforms (i.e. traits in selection indices do not have to be the same as those that are targeted for improvement). Obtaining accurate estimates of variance–covariance matrices is critical as they largely determine the outcome of all subsequent analyses and limit applicability to a specific population and environment. Thus, although the estimation of these matrices from molecular markers is one of the appealing features of this approach (i.e. because conventional methods are much slower and costlier), building in some form of empirical validation would be prudent.

Because the computational cost of evaluating each scenario is very low (i.e. once genetic and phenotypic variance–covariance matrices are estimated), a large set of candidate scenarios can be assessed at the beginning of each breeding cycle. One approach for doing this might be to start from relatively simple scenarios and iteratively modify selection targets and/or economic values as in the examples above. A more systematic alternative might be to perform a search of the multivariate space of scenarios defined by desired gains, correlated responses, economic values and selection intensities using different constraints [e.g. overall cost of the breeding cycle (Gaynor *et al.*, 2017)] or approach the search as an optimization problem (Akdemir and Isidro Sanchez, 2017; Li *et al.*, 2017).

## SUPPLEMENTARY DATA

Supplementary data are available online at https://academic.oup.com/aob and consist of the following. File S1: phenotypic and genetic variance–covariance matrices, correlations and parameter estimates for four genomic index selection scenarios.

mcy187_suppl_aob-18338-s01
